# Coordinating transcription and replication to mitigate their conflicts in early Drosophila embryos

**DOI:** 10.1016/j.celrep.2022.111507

**Published:** 2022-10-18

**Authors:** Chun-Yi Cho, James P. Kemp, Robert J. Duronio, Patrick H. O’Farrell

**Affiliations:** 1 Department of Biochemistry and Biophysics, University of California, San Francisco, San Francisco, CA 94158, USA; 2 Integrative Program for Biological and Genome Sciences, University of North Carolina at Chapel Hill, Chapel Hill, NC 27599, USA; 3 Department of Biology, Department of Genetics, Lineberger Comprehensive Cancer Center, University of North Carolina at Chapel Hill, Chapel Hill, NC 27599, USA; 4 Lead contact

## Abstract

Collisions between transcribing RNA polymerases and DNA replication forks are disruptive. The threat of collisions is particularly acute during the rapid early embryonic cell cycles of *Drosophila* when S phase occupies the entirety of interphase. We hypothesize that collision-avoidance mechanisms safeguard this early transcription. Real-time imaging of endogenously tagged RNA polymerase II (RNAPII) and a reporter for nascent transcripts in unperturbed embryos shows clustering of RNAPII at around 2 min after mitotic exit, followed by progressive dispersal as associated nascent transcripts accumulate later in interphase. Abrupt inhibition of various steps in DNA replication, including origin licensing, origin firing, and polymerization, suppresses post-mitotic RNAPII clustering and transcription in nuclear cycles. We propose that replication dependency defers the onset of transcription so that RNAPII transcribes behind advancing replication forks. The resulting orderly progression can explain how early embryos circumvent transcription-replication conflicts to express essential developmental genes.

## In brief

Early zygotic transcription in *Drosophila* embryos takes place entirely during the short S phases of nuclear division cycles. Cho et al. demonstrate that rapid onset of transcription depends on DNA replication in each cycle. Delayed initiation of transcription allows unimpeded elongation of nascent transcripts behind advancing replication forks.

## Graphical Abstract

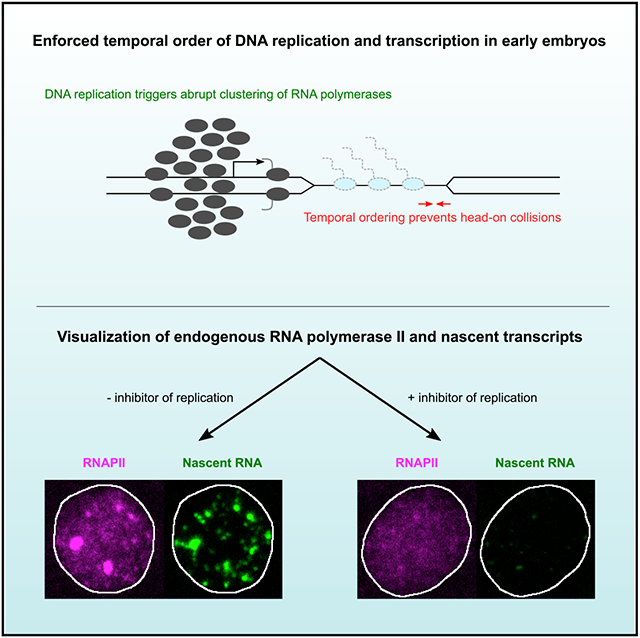

## INTRODUCTION

Reading of the genetic code by both replicative and transcriptional machineries can create conflicts, yet the mechanisms that coordinate template use remain incompletely understood. The expected incompatibility of simultaneous use of the template for replication and transcription was first demonstrated *in vitro* in a T4 bacteriophage system nearly 40 years ago (Bedinger et al., 1983). Collisions, especially head-on collisions between advancing RNA polymerases and replication forks, disrupt both transcription and replication. Such collisions are now known to occur in both prokaryotes and eukaryotes. Bacteria have evolved to co-orient the majority of genes with the direction of replication on their circular genomes, thus minimizing head-on collisions (Merrikh et al., 2012). Cells have also evolved numerous mechanisms to resolve collisions when they do occur and to mitigate the genome instability that can arise (García-Muse and Aguilera, 2016). Yet beyond this, we know little about how replication and transcription might be coordinated to minimize disruptive conflicts.

The *Drosophila* embryo provides an ideal system to probe for mechanisms that mitigate conflicts between replication and transcription. After fertilization, the embryos undergo a series of rapid cleavage cycles, during which nuclei alternate between S phase and mitosis without gap phases. Although transcription is minimal in the first few cycles, a minor wave of gene expression occurs during nuclear cycles (nc) 8–13 before the dramatic cell-cycle slowing in nc14 (Schulz and Harrison, 2019). Effective gene expression during this early wave is highly constrained by the speed of the nuclear cycles. The abortion of nascent transcripts upon entering mitosis leaves a short window for transcriptional elongation in each interphase (Shermoen and O’Farrell, 1991; Strong et al., 2020). Even within interphase, transcription faces the threat of potential collisions with replication forks, which share the same DNA templates and are charged with replicating the entire genome in the same short interphase. How early transcription in embryos is coordinated with DNA replication is unknown.

**Figure 1.**
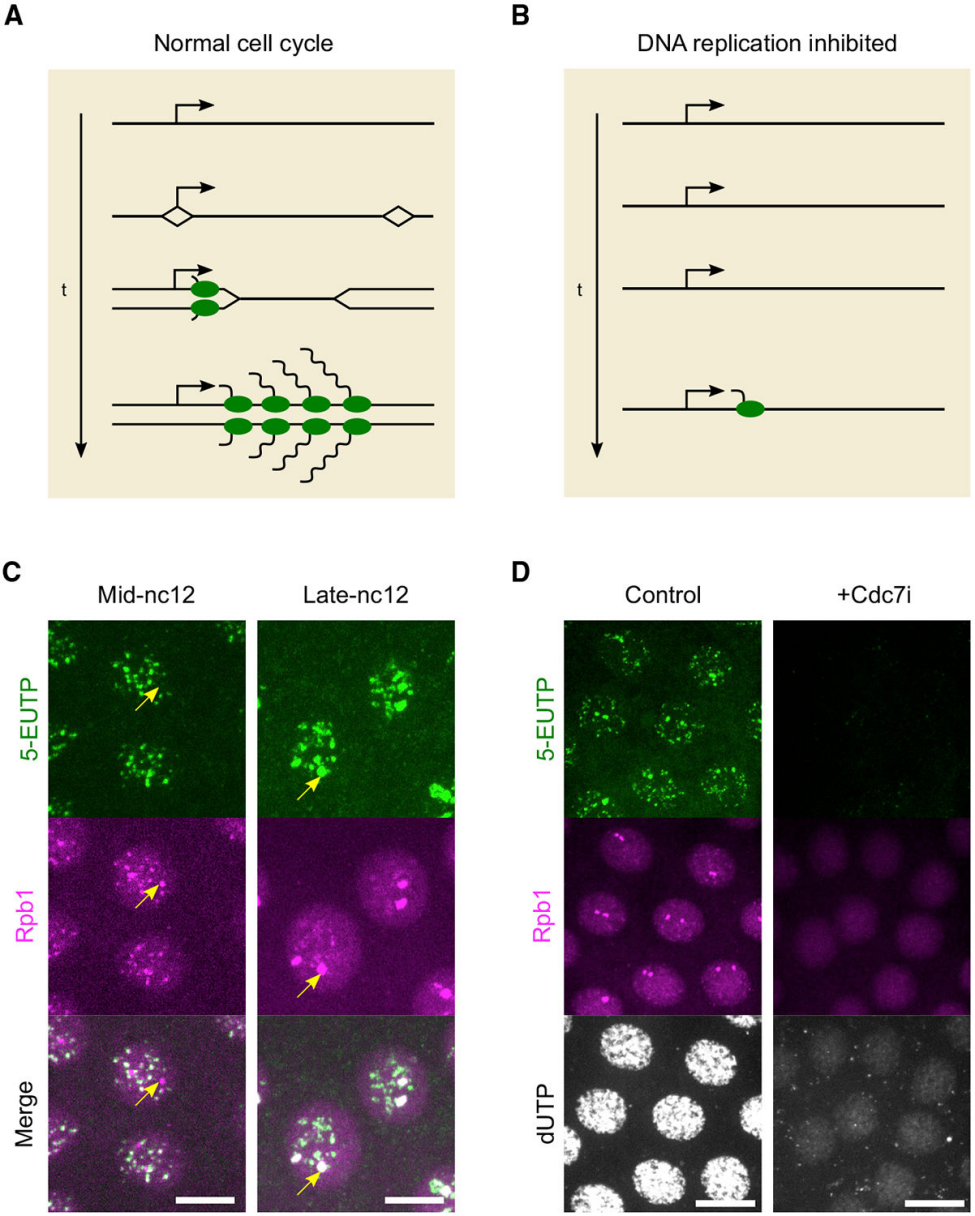
An inhibitor of DNA replication suppresses RNAPII clustering and foci of rNTP incorporation (A) A hypothetical model for the coordination of transcription and DNA replication during rapid cell cycles. In early *Drosophila* embryos, origins are closely spaced throughout the genome. If transcription initiates on DNA not yet replicated, the elongating polymerases and nearby replication forks will rapidly converge in head-on collisions. This problem can be avoided if there is a temporal order of DNA replication and transcription, allowing RNA polymerases to initiate on replicated DNA and follow replication forks. (B) One way to enforce the temporal order is to make onset of transcription dependent on prior DNA replication, which would be experimentally detected as a delay in the onset of transcription upon acute inhibition of DNA replication. (C) Representative images of mCherry-Rpb1 embryos injected with 5-EUTP and fixed in nuclear cycle (nc) 12. The “click” reaction with Alexa Fluor 488 azide was then performed to label 5-EUTP incorporated into nascent transcripts. The stages in interphase were determined by the sizes of nuclei and large Rpb1 clusters. Arrows point to examples of large Rpb1 clusters associated with histone locus bodies. Scale bars, 6 μm. (D) Representative images of mCherry-Rpb1 embryos injected during mitosis 11 with water (control) or Cdc7i along with 5-EUTP and Cy5-dUTP and fixed at about 7 min in nc12. Scale bars, 12 μm. Similar outcomes were observed in 3 embryos. See also Figure S1.

In the early cell cycles without gap phases, DNA replication begins immediately following mitosis, and so its onset precedes that of transcription about 3 min later (Figure 1A) (Blumenthal et al., 1974; Garcia et al., 2013; Lucas et al., 2013; McCleland et al., 2009; Shermoen et al., 2010). Since DNA replication forks and RNA polymerases move at a similar speed in eukaryotes (Shermoen and O’Farrell, 1991; Yeeles et al., 2017), collisions could be avoided if RNA polymerase II (RNAPII) initiates only on replicated sections of DNA, allowing transcriptional elongation to follow replication forks. This led us to hypothesize the existence of a coupling mechanism that would enforce a delay in transcription until the initiation of replication (Figure 1B).

Here, we show that DNA replication is required for the rapid onset of transcription after mitosis in each nuclear cycle. We used live microscopy to track the clustering of endogenously tagged RNAPII at sites of transcription and to follow the emergence of tagged nascent transcripts. Acute inhibition of DNA replication by three inhibitors with different molecular targets consistently impaired both RNAPII clustering and nascent transcript synthesis. Our data suggest that the abrupt clustering of RNAPII promotes a “burst” of transcriptional initiation that follows the onset of DNA replication. The coupling of transcription to replication can ensure that few, if any, RNA polymerases occupy DNA templates ahead of replication forks, thereby minimizing collisions.

## RESULTS

### An inhibitor of replication suppresses RNAPII clustering and foci of rNTP incorporation

We used CRISPR-Cas9 to tag the N-terminal end of two RNAPII subunits, Rpb1 (*Polr2A*) and Rpb3 (*Polr2C*), with mCherry or EGFP, respectively. Despite a slightly lower hatch rate of embryos laid by homozygous EGFP-Rpb3 females, flies homozygous for either mCherry-Rpb1 or EGFP-Rpb3 are healthy and fertile (Figure S1A). In embryos co-expressing the two markers, the nuclear foci of Rpb1 and Rpb3 co-localized (Figure S1B), indicating that foci represent clusters of RNAPII complexes rather than aggregates of fluorescent proteins.

Clusters of RNAPII are expected to mark sites of expressed loci (Boehning et al., 2018; Cho et al., 2016, 2018; Cisse et al., 2013; Huang et al., 2021; Pancholi et al., 2021). To confirm transcriptional activity at RNAPII clusters, we injected 5-ethynyl-uridine-5’-triphosphate (5-EUTP) into embryos expressing mCherry-Rpb1 and then fixed the injected embryos for labeling with Alexa Fluor 488 azide via a “click” reaction (STAR Methods). In nc12 embryos, 5-EUTP was incorporated into bright foci that co-localized with mCherry-Rpb1 foci, consistent with localized RNA synthesis by RNAPII (Figure 1C). As previously seen (Huang et al., 2021), two classes of RNAPII clusters were apparent in each nucleus (Figure S1B). The numerous dispersed small clusters were distinct from one or two large clusters at the histone locus bodies (HLBs) identified by co-staining with mScarlet-tagged Mxc (Figure S1C) (Kemp et al., 2021; Terzo et al., 2015). These HLB signals become especially dominant later in interphase (e.g., Figure 1C).

To test whether DNA replication impacted the onset of transcription after mitosis, we co-injected embryos with 5-EUTP and an inhibitor of Cdc7, the catalytic subunit of an S-phase kinase that triggers origin firing (Sheu and Stillman, 2010). As expected, co-injection of this inhibitor, XL413 or Cdc7i herein (Cho et al., 2022; Koltun et al., 2012; Stephenson et al., 2015), during mitosis 11 greatly reduced incorporation of dUTP into DNA in the following nc12 (Figure 1D), supporting a strong inhibition of DNA replication. Surprisingly, the inhibitor also suppressed incorporation of 5-EUTP and the clustering of mCherry-Rpb1 in nc12 (Figures 1D and S1D). We conclude that, in addition to being required for robust DNA replication, Cdc7 contributes to the formation of RNAPII clusters and global RNA synthesis.

### RNAPII forms dynamic clusters that transiently co-localize with nascent transcripts

**Figure 2.**
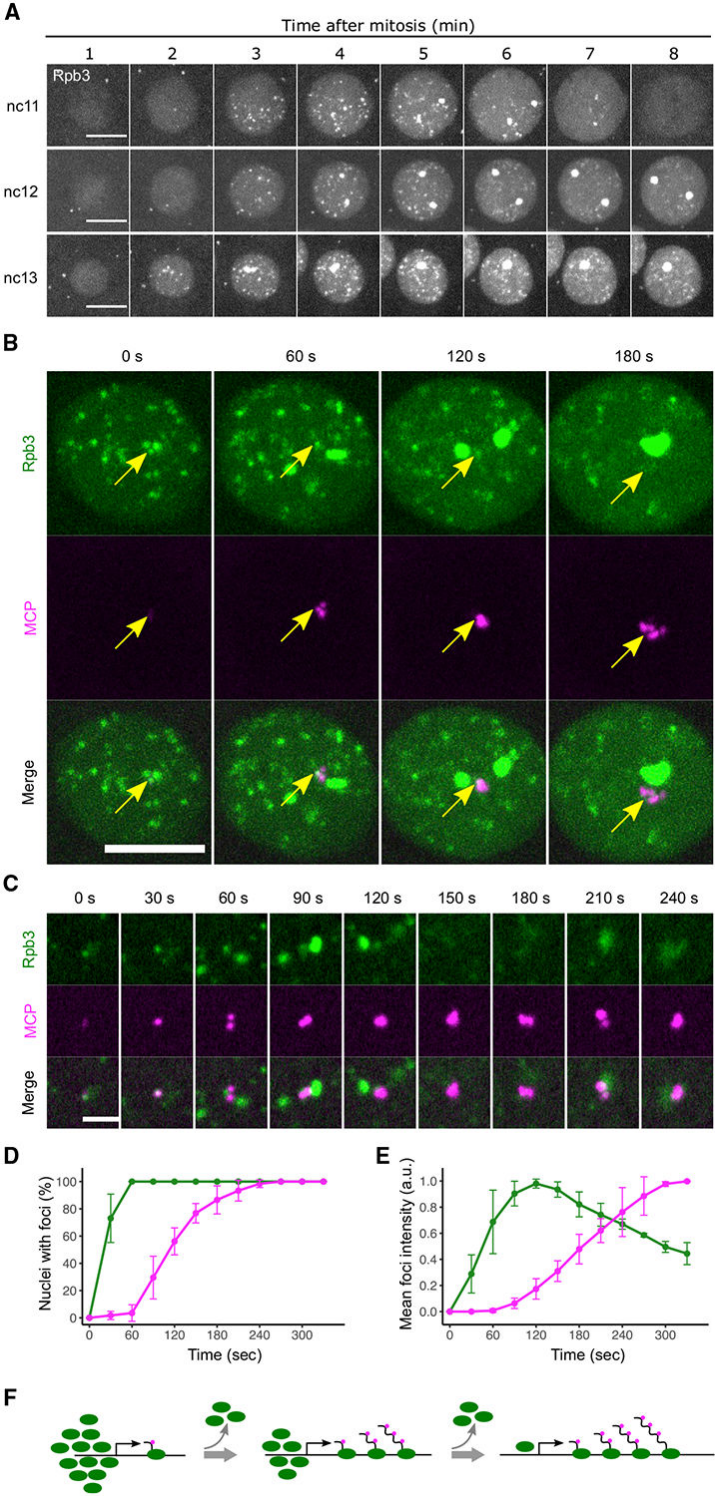
RNAPII clusters transiently co-localize with nascent transcripts (A) Representative stills from live imaging of endogenously tagged EGFP-Rpb3 during nc11–13. Maximal projections across the entire nuclei are shown. In each cycle, images centering on a single nucleus are cropped and displayed. The 0 min time point is determined manually using His2Av-mRFP as shown in Figure S1F. (B) Representative stills from live imaging of EGFP-Rpb3 and MCP-mCherry in an embryo carrying the *hbP2-MS2-lacZ* reporter during early nc12. Maximal projections of the entire nucleus are shown. The 0 s time point refers to the frame when the MCP focus first appeared. Similar outcomes were observed in 10 embryos. Scale bar, 5 μm. (C) Single focal planes focusing on the same MCP spot from (B). The 0 s time point refers to the frame when the MCP focus first appeared. A very weak MCP focus at the beginning of the sequence aligns with an Rpb3 focus (central), and the large HBL focus of Rpd3 develops next to it. The imaging plane remains on the MCP focus, and the intensity of a nearby HLB-associated Rpb3 cluster varies as it moves in (e.g., 90 s frame) and out of the focal plane (e.g., 150 s frame). Scale bar, 2 μm. (D) Percentages of nuclei with RNAPII clusters (green) or MCP foci (magenta) during nc12. The 0 s time point refers to the frame before RNAPII cluster formation. Error bars represent SD, n = 3 embryos. (E) Mean intensity of all non-HLB RNAPII clusters (green) or MCP foci (magenta) during nc12. The 0 s time point refers to the frame before RNAPII cluster formation. Error bars represent SD, n = 3 embryos. (F) A model for the relationship between RNAPII clusters (green) and nascent transcripts (MCP, magenta) during activation of the transcription unit. See also Figures S2.

Next, we used live microscopy to investigate the impact of replication on the onset of transcription in more detail. We first characterized the clustering of fluorescently tagged RNAPII and examined its relationship to the expression of a transcriptional reporter during unperturbed nuclear cycles (Figure 2).

We detected sparse RNAPII clusters as early as nc10 when nuclei migrated from the interior of the embryo to the surface shortly after the beginning of zygotic gene expression (Figure S1E). In each of the remaining syncytial blastoderm cycles (nc11–13), RNAPII initially showed a uniform distribution in the nucleus for the first 1–2 min, followed by the abrupt formation of clusters at 2–4 min (Figure 2A). The two classes of RNAPII clusters had distinct cell-cycle dynamics in addition to having different sizes (Figures 2A and S1F). In nc11, the small and dispersed clusters emerged around 2 min, peaked in number and intensity 1–2 min later, and then declined through the remainder of interphase (Figure 2A). Roughly at the time of the peak intensity of the dispersed clusters, the large clusters at HLBs became apparent. These HLB-associated clusters increased in size throughout interphase, persisted through prophase, and declined in prometaphase. The distinction in the dynamics of the two classes became more dramatic in nc12 and nc13. Different ratios of Rpb1 and 5-EUTP incorporation seen in fixed embryos also suggested this distinct dynamic for HLB and non-HLB clusters of RNAPII (Figure 1C). These distinctions in the kinetics of HLB and non-HLB foci may be related to other known differences. For example, transcription of many of the early wave genes depends on the transcription factor Zelda, while the histone genes have distinct inputs (Huang et al., 2021; Hur et al., 2020; Liang et al., 2008).

To determine the relationship between the formation of RNAPII clusters and the synthesis of nascent transcripts, we introduced the MS2/MCP nascent transcript live-imaging system into EGFP-Rpb3 embryo and focused on a synthetic reporter for the gap gene *hunchback* (*hb*). The *hbP2-MS2-lacZ* construct contains the *hb* enhancers and *hbP2* promoter and is broadly active in the anterior half of the embryo after nc9 (Garcia et al., 2013). In a normal nc12, all nuclei first acquired RNAPII clusters relatively synchronously about 2 min after mitosis, while the transcription of the *hbP2-MS2* reporter in anterior nuclei (detected by MCP-mCherry) was more stochastic and appeared in different nuclei between 2.5 and 5 min after mitosis (Figures 2B, 2D, and S2A). In 90% of anterior nuclei (60/67 from 3 embryos), we detected a single MCP focus that emerged in apparent association with a small RNAPII cluster (Figures 2B and 2C, 0 s frames). Overlap of the MCP and Rpb3 signals initially persisted but was dynamically rearranged, which is more clearly seen in single-focal-plane images (Figure 2C). The Rpb3 cluster simply persisted while the MCP signal initially emerged (Figures 2C, 0–30 s). Shortly after, disruptions in the MCP signal and the Rpb3 cluster complicate comparison, but Rpb3 signal intensity appeared to decline, while the MCP signal increased dramatically (Figures 2C, 30–120 s). While the MCP focus remained at high intensity for much of the rest of interphase, the Rpb3 cluster faded and was barely distinguishable in later interphase (Figures 2B and 2C, after 120 s).

Because the *hbP2-MS2* reporter is closely linked to the *HisC* (histone gene) locus, in some frames of the videos, the high HLB-associated EGFP-Rpb3 signal obscured the EGFP signal associated with the reporter. This feature interfered with quantification of the reporter-associated RNAPII over time. To portray the dynamics of the RNAPII clusters, we quantified the entire population of small clusters and compared this with *hbP2-MS2* dynamics during nc12 (Figure 2E). The average intensity of small RNAPII clusters peaked about 2 min after their initial emergence (Figure 2E). Intensity then declined for the rest of interphase. In contrast, the intensity of MCP foci for *hbP2-MS2* only started to increase as the average intensity of RNAPII clusters peaked. The MCP foci reached high intensity in late interphase, when most RNAPII clusters were much reduced or almost undetectable (Figures 2B, 180 s frame, and 2E). We observed a similar outcome using a separate line expressing mCherry-Rpb1 and MCP-GFP (Figure S2B). Furthermore, comparison of 5-EUTP incorporation with mCherry-Rpb1 clusters in embryos fixed later in interphase similarly showed that, globally, the RNAPII clustering was reduced in later interphase, while the nascent transcript levels were increased (Figure 1C).

Taken together, these observations support the association of RNAPII clusters with nascent transcripts. However, they also indicate that accumulation of nascent transcripts is not paralleled by expansion of RNAPII clusters. We suggest that the abrupt and early formation of RNAPII clusters represents a distinct form of recruitment to increase the local concentration of RNAPII. The subsequent maturation and early reductions of RNAPII clusters suggest that only a minority of RNAPII recruited to the initial cluster contributes to the pool of transcribing polymerases (Figure 2F).

### The abrupt formation of RNAPII clusters and prompt expression of a transcriptional reporter require DNA replication

**Figure 3.**
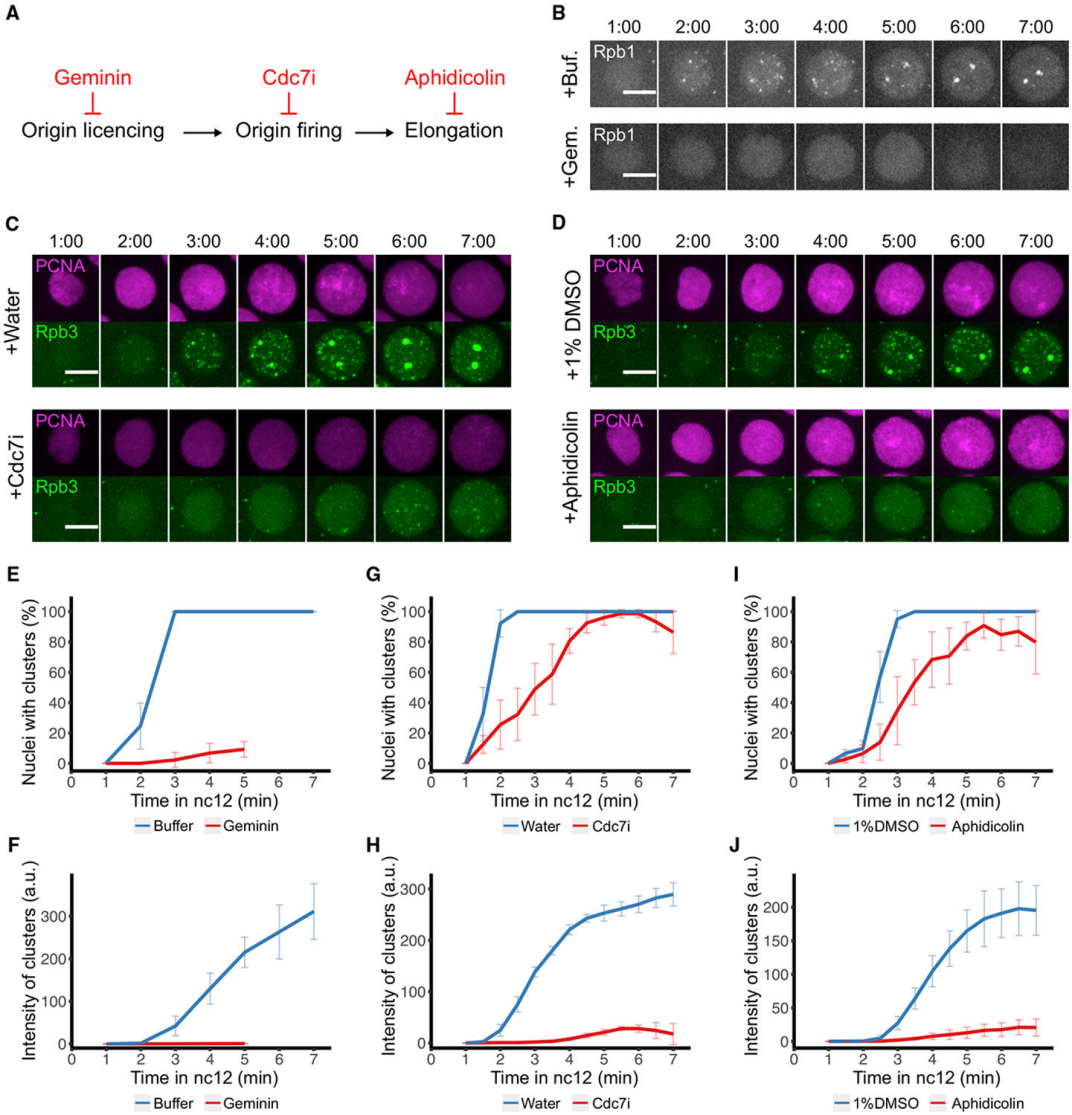
DNA replication is required for the abrupt formation of RNAPII clusters after mitosis (A) Diagram illustrating different steps in DNA replication and corresponding inhibitors. In all experiments in this figure and Figure 4, the injections of Cdc7i and aphidicolin were performed during mitosis, and the following interphase was recorded by confocal live imaging. Control and Geminin injections were performed during the previous interphase or mitosis, which yielded similar results. (B) Representative stills from live imaging of mCherry-Rpb1 in embryos injected with microinjection buffer as control or 20 mg/mL ECFP-Geminin (not showing here). The 0 min time point is set as the first frame with nuclear mCherry-Rpb1 signal upon entering nc12. (C) Representative stills from live imaging of mCherry-PCNA, which marks replicating loci, and EGFP-Rpb3 in embryos injected with water or 0.5 mg/mL Cdc7i. The 0 min frame (not shown) was set as the first frame with visible chromatin-bound PCNA in nc12. (D) Representative stills from live imaging of mCherry-PCNA and EGFP-Rpb3 in embryos injected with 1% DMSO as control or 0.1 mg/mL aphidicolin in 1% DMSO. The 0 min frame (not shown) was set as the first frame with visible chromatin-bound PCNA in nc12. (E, G, and I) Percentages of nuclei having RNAPII clusters (using either mCherry-Rpb1 or EGFP-Rpb3) during nc12 in embryos after indicated injections. The experimental data for Geminin injection are truncated because this inhibitor shortens interphase. Error bars represent SD, n R ≥ embryos. (F, H, and J) Total fluorescent intensity of RNAPII clusters per nucleus in embryos after indicated injections. Error bars represent SD, n ≥ 3 embryos. All scale bars, 5 μm. See also Figure S3.

**Figure 4.**
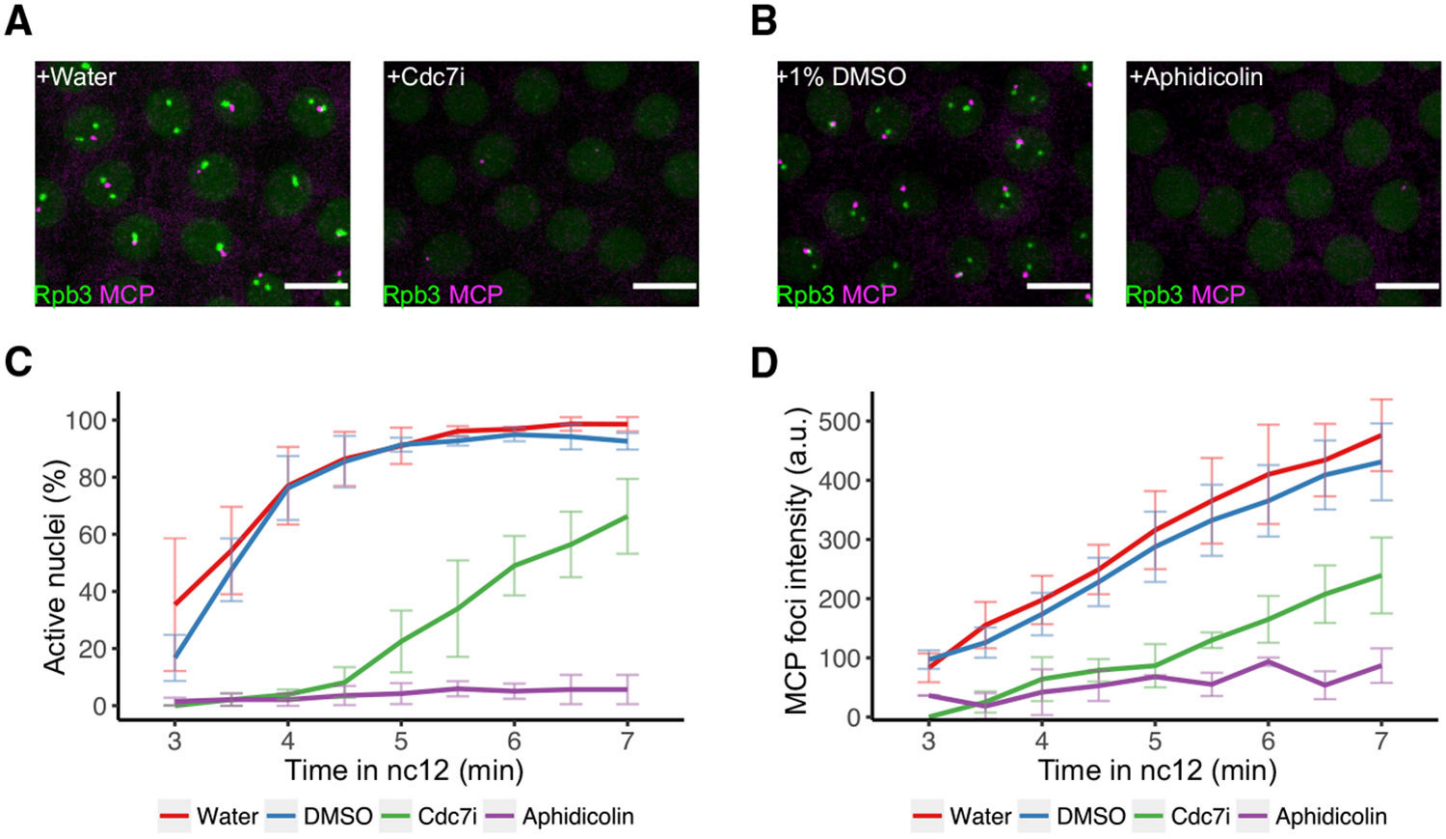
DNA replication is required for the rapid onset of transcription after mitosis (A and B) Representative stills from live imaging of EGFP-Rpb3 and MCP-mCherry in embryos carrying the *hbP2-MS2-lacZ* reporter after indicated injections. The 0 min time point is set as the first frame with visible nuclear EGFP-Rpb3 signal, and frames at the 6 min are shown. Scale bars, 5 μm. (C) Percentages of nuclei having MCP foci in embryos carrying *hbP2-MS2* reporter during nc12 after indicated injections. Error bars represent SD, n = 3 embryos. (D) Mean intensity of emerged MCP foci in embryos carrying *hbP2-MS2* reporter during nc12 after indicated injections. Error bars represent SD, n = 3 embryos.

Using real-time measures of RNAPII cluster formation and the nascent transcript reporter, we tested more fully the impact of inhibitors of DNA replication on the onset of transcription (Figures 3 and 4).

Eukaryotic DNA replication begins with the licensing of origins. We inhibited one of the licensing factors, Cdt1/Dup, by injecting its protein inhibitor Geminin (Figure 3A) (McCleland et al., 2009). The injection of purified Geminin in one cycle blocks the licensing of origins for the next cycle. Geminin injection in nc11 abolished the formation of Rpb1 clusters in nc12, including both the large clusters at HLBs and the many small clusters throughout the nucleus (Figure 3B). Only a few nuclei exhibited sparse and very faint Rpb1 clusters toward late nc12 (Figures 3E and 3F). In the absence of licensed DNA, S phase is effectively deleted, and there is no signal to activate the DNA replication checkpoint that normally lengthens pre-MBT cycles (Fogarty et al., 1997; Sibon et al., 1997). As a result, syncytial blastoderm embryos progress through a shortened interphase (Figure S3A) and enter mitosis 12 with unreplicated and uninemic chromosomes (McCleland et al., 2009). Similar abolishment of Rpb1 clusters was observed in nc13 when Geminin was injected in nc12 (Figure S3A).

Following origin licensing and the formation of pre-replication complexes (pre-RCs), S-phase kinases activate MCM helicases in the pre-RCs to trigger origin firing. As described above, we injected Cdc7i to suppress this step (Figures 1D and 3A). In real-time imaging, Cdc7i suppressed the recruitment of mCherry-PCNA, which reports the formation of replication forks (Seller and O’Farrell, 2018), and delayed the abrupt formation of RNAPII clusters in nc12 (Figures 3C and S3B). While RNAPII clusters were eventually observed in most nuclei, their appearance was delayed, their intensity was reduced, and the large clusters at HLBs failed to form (Figures 3C, 3G, and 3H). The low level of dUTP incorporation (Figure 1D) along with other studies (Cho et al., 2022; Suski et al., 2022) indicate that Cdc7 inhibition only reduced the frequency of origin firing and so slowed the accumulation of replication forks. Thus, the delayed and reduced RNAPII cluster formation might result from incomplete inhibition of DNA replication. Alternatively, it might represent a slower and secondary pathway for RNAPII clustering independently of DNA replication. We conclude that Cdc7 activity, or some Cdc7-dependent event such as DNA replication, stimulates the initial abrupt formation of RNAPII clusters following mitosis.

We injected embryos with a third specific inhibitor of DNA replication, aphidicolin, which is a competitive inhibitor of dNTP used by DNA polymerase alpha (Figure 3A). Forks still form in the presence of aphidicolin, but the rate of their progression is quantitatively slowed by the drug. Such inhibited forks persist and induce checkpoint activation, and nuclei enter a delayed mitosis followed by anaphase bridges (Sibon et al., 1997). Consistent with this, mCherry-PCNA signal persisted through late interphase without resolving (Figure 3D, bottom panel, 7 min frame), and mitosis was delayed (Figure S3C). In further support of a dependency of transcription on replication, the emergence of RNAPII clusters was nearly abolished by aphidicolin injection (Figures 3D, 3I, 3J, and S3C). The finding that three specific inhibitors of replication, each acting at distinct steps in the process, prevent early post-mitotic RNAPII clustering leads us to conclude that this prompt clustering is directly or indirectly dependent on DNA replication.

Finally, we tested the effects of Cdc7i and aphidicolin injections on the expression of *hbP2-MS2* reporter (Figure 4). Both inhibitors delayed and suppressed *hbP2-MS2* expression (Figures 4A and 4B). In Ccd7i-injected embryos, the percentage of active nuclei increased later and more slowly in late interphase (Figure 4C), but once emerged, the intensity of those MCP foci appeared to increase at a rate comparable to control (Figure 4D). Given the action of this inhibitor on the firing of origins, these observations could be explained by a scenario in which RNAPII recruitment awaits the passage of infrequently initiated forks but then transcribes behind normally progressing forks. Injection of aphidicolin led to a more stringent block of both RNAPII clustering and transcription throughout interphase (Figure 4B). Only a few nuclei exhibited faint MCP foci (Figure 4C), whose intensity stayed low throughout our imaging (Figure 4D). These observations could be explained by a scenario in which extension of transcripts initiated on rare amounts of replicated DNA during aphidicolin treatment is slowed because they are following sluggishly moving forks. Taken together, we conclude that DNA replication is required for both timely RNAPII clustering and transcriptional initiation of the *hbP2-MS2* reporter.

## DISCUSSION

Our findings herein show that the onset of post-mitotic transcription during early embryonic cell cycles is linked to prior DNA replication, an arrangement that can allow collision-free overlap of transcription and replication with engaged RNAPII following replication forks. This coordination of transcription with replication is particularly crucial during the extremely short interphases of the early *Drosophila* embryo when the two processes necessarily overlap.

The regulation we describe herein requires two components. First, there must be some constraint that limits transcription on early post-mitotic chromatin. Second, some aspect of replication must reverse or override this constraint. While unknown, we speculate that DNA replication, by promoting decompaction (McCleland et al., 2009; Sonneville et al., 2015) and modification of chromatin, accelerates the reversal of transcriptionally quiescent mitotic chromatin to give a timely burst of transcriptional initiation. Whatever the input of replication might be in promoting transcription, given the key role of the transcription factor Zelda in activating transcription in the early fly embryo (Huang et al., 2021; Liang et al., 2008; McDaniel et al., 2019), we expect that it will operate in conjunction with Zelda, perhaps by promoting re-establishment of interphase chromatin loops and enhancer-promoter contacts (Espinola et al., 2021; Ogiyama et al., 2018).

We expect coordination to be widely important in somatic cells with longer S phase. In the absence of coordination, ongoing transcription of unreplicated DNA will lead to inevitable collisions when replication forks advance into transcribed regions. Perhaps suggesting control similar to what we describe here, recent work in the mammalian cell cycle indicates that even though the known limitations for transcription of replication-dependent histone genes are satisfied at the restriction point, full activation of their transcription still awaits the onset of replication (Armstrong and Spencer, 2021). Regardless of the details, the clear coupling of transcription to replication in the early *Drosophila* embryo is likely to provide a system in which we can work out mechanisms that can avoid, rather than those simply resolve, the problems caused by simultaneous use of the DNA template for both replication and transcription.

### Limitations of the study

While the data document a coupling of transcription to replication, they neither tell us how or why this coupling occurs. A test of the potential role in collision avoidance has to await a means of manipulating the coupling, which requires an understanding of its mechanism. At present, we do not know what prevents an immediate transcription following mitosis and what kind of input DNA replication provides to promote its eventual onset. The ability of aphidicolin to suppress transcription suggests that replication and/or fork progression is essential, but earlier steps in the initiation of replication might contribute additional inputs to activate transcription.

## STAR★METHODS

### RESOURCE AVAILABILITY

#### Lead contact

Further information and requests for resources and reagents should be directed to the lead contact, Patrick H. O’Farrell (ofarrell@cgl.ucsf.edu).

#### Material availability

Fly stocks generated in this study are available from the lead contact upon request.

#### Data and code availability

- Raw imaging data have been deposited at Zenodo and is publicly available as of the date of publication. The DOI is listed in the key resources table.
- All original codes have been deposited at Zenodo and is publicly available as of the date of publication. The DOI is listed in the key resources table.
- Any additional information required to reanalyze the data reported in this paper is available from the lead contact upon request.

### EXPERIMENTAL MODEL AND SUBJECT DETAILS

All experiments were performed on *Drosophila melanogaster*. Fly stocks were maintained on standard cornmeal-yeast medium at 25°C. Flies were transferred to egg-laying cages 2-3 days before experiments, and embryos were collected on grape juice agar plates with yeast pastes. Young embryos obtained from adult females within two weeks of eclosion were used for live microscopy. Fly lines used in this study are listed in the key resources table.

### METHOD DETAILS

#### Molecular cloning and CRISPR-Cas9 genome editing

Oligos for sgRNA targeting 5’ ends of Rpb1 and Rpb3 were ordered from IDT, annealed and cloned into pU6-BbsI-chiRNA using standard protocol (Gratz et al., 2013). To make donor plasmids, about 1 kb of homology arms upstream and downstream of start codons were amplified from genomic DNA of the *vas-Cas9* flies. EGFP and mCherry DNA fragments with 5xGGS linker were amplified from plasmids previously made in the lab. The pDsRed-attP was cut with XhoI and HindIII to be used as vector backbone. All DNA fragments were then purified by gel purification and assembled by Gibson assembly. The sgRNA and donor plasmids were sent to Rainbow Transgenic Flies (Camarillo, CA) for microinjection. After injection, surviving adults were crossed to appropriate balancer stocks and screened by PCR for successful knock-in. Transformants were backcrossed with wild type at least three times before performing experiments.

#### Hatch rate assay

Embryos were collected for 2-4 h in egg-laying cages, washed with water, and transferred to grape juice agar plates. 50 embryos in each assay were aligned in groups of 10. Percentages of embryos hatched were then scored after 36 h of incubation at 25°C.

#### Spinning disk confocal microscopy

Imaging of live or fixed embryos was performed on an Olympus IX70 microscope equipped with PerkinElmer Ultraview Vox confocal system. All experiments were performed at room temperature with 63x or 100x oil objective, and binning was set to 1x1 or 2x2 depending on the experiments. Data were acquired using Volocity 6 software (Quorum Technologies). Fluorophores were excited with 488, 561, and 640 nm laser lines and imaged with proper emission filters. Focal planes each 0.5–0.75 μm apart were recorded at each timepoint, spanning 3–10.5 μm across the nuclei at the surface of the embryos. Images in the same set of experiments were acquired using the same configuration, and laser power was calibrated using a laser power meter (Thorlabs) before each imaging session.

#### Embryo mounting for live imaging

Embryos were collected in egg-laying cages, washed and transferred into a basket, dechorionated with 25% bleach for 2 min, washed three times with water, and transferred onto grape juice agar plates. Embryos were aligned and transferred to coverslips with glue derived from double-sided tape using heptane. The embryos were then covered with 1:1 mixture of halocarbon oil 27 and 700.

#### Microinjection

After aligned and glued to a coverslip, embryos were desiccated in a desiccation chamber for 7-9 min before being covered in halocarbon oil for microinjection. XL413 was dissolved in water at 5 mg/mL and stored at −20°C as stock solution. Aphidicolin was dissolved in DMSO at 10 mg/ml as stock solution. Injections were performed at the following concentrations: 0.5 mg/mL XL413/Cdc7i, 0.1 mg/mL aphidicolin (in 1% DMSO), and 20 mg/mL ECFP-geminin (in 40 mM HEPES pH 7.4, 150 mM KCl buffer).

#### Fly crosses for MS2/MCP imaging

Virgins carrying endogenously tagged RNAPII subunits and expressing tagged MCP in the germline were crossed with males of *hbP2-MS2-lacZ* for 2-3 days. Flies were then transferred together into a cage, and embryos laid were collected for imaging. All crosses and embryo collection were performed at 25°C.

#### 5-EUTP labeling for RNA imaging

##### Injection and fixation

Our preliminary data with 5-EU suggest it takes longer (>15 min) to be incorporated into transcripts and labels predominantly rRNA transcripts. 5-EUTP was thus used for faster and more efficient labeling of RNAPII transcription. For the collection of each embryo, about 6 embryos from mCherry-Rpb1 females were manually dechorionated on double-sided tape, desiccated, and covered in halocarbon oil as described above. Live confocal microscopy was used for staging, and a single embryo entering mitosis 11 was identified by the release of mCherry-Rpb1 from the nucleus into cytoplasm when nuclear membrane breaks down. The embryo was injected with a mixture of 10 mM 5-ethynyl-uridine-5’-triphosphate (5-EUTP) +/− 1 mg/mL XL413 in water in mitosis 11. Cy5-dUTP was co-injected at 0.1 mM in a subset of embryos to confirm inhibition of DNA replication. After injection of targeted embryo, the rest of non-injected embryos were removed along with excess halocarbon oil. The injected embryo was incubated at room temperature until 12 min after entry into mitosis 11 (approximately 7 min in nc12). The embryo was washed off the glue by heptane, transferred to a 20-mL scintillation, and fixed by a 1:1 mixture of heptane and 37% formaldehyde (5 mL each). Fixation was done with rigorous shaking for 1 min and further standing for 11 min at room temperature. After fixation, the bottom layer of formaldehyde was removed. Equal volume of methanol was then added, followed by rigorous shaking for 1 min. The top layer of heptane was removed as much as possible, and then fresh methanol was added until the embryo sank from the interface to the bottom. The supernatant was replaced with fresh methanol once, and the sample was stored at −20°C until enough samples have been collected.

To manually remove the vitelline membrane, a glass dish was prepared with a strip of double-sided tape and covered with 1 mL PBS. Embryos were first transferred with glass pipette onto a nylon mesh, washed three times with PBS, then transferred into PBS on the double-sided tape. Embryos were gently rolled on the tape to remove the vitelline membrane. 4 mL PBST (0.3% tween 20) was then added to collect the embryos and transfer them to a 0.5-mL tube.

##### Click reaction for Alexa Fluor 488 labeling

Embryos were washed with PBST for 5 min four times. The “click” reaction was then performed following manufacturer’s protocol in a 0.5-mL reaction for 30 min. The embryos were then rinse once with the Click-iT reaction rinse buffer, washed with PBST for 10 min twice, and mounted on a glass slide in Fluoromount. The fluorescence of mCherry was largely preserved after fixation and click re-action, so we directly imaged the endogenous fluorescence of mCherry-Rpb1 along with 5-EUTP/Alexa488 without immunostaining.

#### Image processing and presentation

Data obtained in Volocity were exported as image stacks and processed in FIJI/ImageJ (Schindelin et al., 2012) and Python. Maximal projections are shown in figures unless otherwise noted.

### QUANTIFICATION AND STATISTICAL ANALYSIS

#### Quantification of RNAPII and MCP foci

##### Image segmentation

The segmentation of RNAPII clusters and MCP foci was performed by the Trainable Weka Segmentation tool in FIJI (Arganda-Carreras et al., 2017). Images were maximally projected and background-subtracted using the rolling ball method. Selected frames from control embryos were used to train the “FastRandomForest” classifier with the default parameters. The classifier was then applied to all images. For quantification of total RNAPII clusters (Figure 3), images were segmented into all RNAPII clusters (both small and large clusters) and background (cytoplasm and nucleoplasm). For quantification of non-HLB RNAPII clusters (Figure 2), images were segmented into small clusters and background (cytoplasm, nucleoplasm, and large clusters). For quantification of MCP foci, images were segmented into foci and background.

The segmentation of nuclei was performed by Otsu’s thresholding following Gaussian blurring in FIJI. For inhibitor-injected embryos (Figure 3), mCherry-Rpb1 or mCherry-PCNA images were used. For MS2 imaging (Figures 2 and 4), EGFP-Rpb3 images were used.

##### Quantification

Raw images were first background-subtracted by the rolling ball method and quantified using masks from segmentation results in Python. The nucleoplasmic intensity in each image was determined as the median of nuclear intensity and subtracted from all pixels before quantification of the intensity of foci.

#### Quantification of 5-EUTP intensity

Images of mCherry-Rpb1 were used for nuclear segmentation as described above. Images of 5-EUTP were background-subtracted by the rolling ball method and then quantified for mean fluorescent intensity within or outside nuclei. The difference between mean nuclear and cytoplasmic intensity was then scored for each embryo.

#### Statistical analysis

All experiments in this study were repeated at least three times with similar results. Representative images from one experiment are shown, and mean ± SD (n ≥ 3 embryos) are plotted. One-tailed Student’s t-test was performed in R (v.3.2.1) to determine the difference of embryonic hatch rates. A value of p < 0.05 was considered statistically significant. Additional details can be found in the corresponding figure legends.

## Supplementary Material

1

## Figures and Tables

**Table:** KEY RESOURCES TABLE

REAGENT or RESOURCE	SOURCE	IDENTIFIER
Chemicals, peptides, and recombinant proteins		
Halocarbon oil 27	Sigma-Aldrich	Cat#H8773
Halocarbon oil 700	Sigma-Aldrich	Cat#H8898
XL413 hydrochloride	Sigma-Aldrich	Cat#SML1401; CAS#1169562-71-3
Aphidicolin from Nigrospora sphaerica	Sigma-Aldrich	Cat#A0781; CAS#38966-21-1
ECFP-Geminin	(McCleland et al., 2009)	N/A
5-EUTP	Abcam	Cat#ab146744
Cy5-dUTP	Sigma-Aldrich	Cat#GEPA55022
Fluoromount aqueous mounting medium	Sigma-Aldrich	Cat#F4680
37% formaldehyde	Thermo Fisher Scientific	Cat#F79-500
Deposited data		
Raw imaging data	This paper; Zenodo	Zenodo: https://doi.org/10.5281/zenodo.7102432
Custom codes	This paper; Zenodo	Zenodo: https://doi.org/10.5281/zenodo.7102452
Experimental models: Organisms/strains		
*D. melanogaster*: Wild type. *Canton S w[1118]*	This paper	N/A
*D. melanogaster*: *w*; *EGFP-Rpb3*	This paper	N/A
*D. melanogaster*: *w*, *mCherry-Rpb1*	This paper	N/A
*D. melanogaster*: *w*, *mCherry-Rpb1*; *EGFP-Rpb3*	This paper	N/A
*D. melanogaster*: *w*; ; *His2Av-mRFP*	Bloomington Drosophila Stock Center	BDSC:23650
*D. melanogaster*: *w*; *EGFP-Rpb3*; *His2Av-mRFP*	This paper	N/A
*D. melanogaster*: *w*; ; *mCherry-PCNA*	(Seller and O’Farrell, 2018)	N/A
*D. melanogaster*: *w*; *EGFP-Rpb3*; *mCherry-PCNA*	This paper	N/A
*D. melanogaster*: *yw*, *Mxc-mScarlet*	(Kemp et al., 2021)	N/A
*D. melanogaster*: *yw*, *Mxc-mScarlet*; *EGFP-Rpb3*	This paper	N/A
*D. melanogaster*: *nos-MCP-mCherry (III)*	Laboratory of Hernan G. Garcia	N/A
*D. melanogaster*: *w*; *EGFP-Rpb3*; *nos-MCP-mCherry*	This paper	N/A
*D. melanogaster*: *nos-MCP-EGFP (II)*	Bloomington Drosophila Stock Center	BDSC:63821
*D. melanogaster*: *w*, *mCherry-Rpb1*; *nos-MCP-EGFP*	This paper	N/A
*D. melanogaster*: y[1] w[*];	Bloomington Drosophila	BDSC:60338
*P{hbP2-MS2-lacZ}JB38F*	Stock Center	
*D. melanogaster*: *w[1118]*; *PBac{y[*+*mDint2]*	Bloomington Drosophila	BDSC:51324
*GFP[E.3xP3]=vas-Cas9}VK00027*	Stock Center	
Oligonucleotides		
Forward strand oligo for Rpb1 sgRNA: CTTCGTCCTGGTCGTCAGCGATAC	This paper	N/A
Reverse strand oligo for Rpb1 sgRNA: AAACGTATCGCTGACGACCAGGAC	This paper	N/A
Forward strand oligo for Rpb3 sgRNA: CTTCGCGGACGGCTGGTTGGCGTA	This paper	N/A
Reverse strand oligo for Rpb3 sgRNA: AAACTACGCCAACCAGCCGTCCGC	This paper	N/A
Forward primer for mCherry-Rpb1 upstream homology arm: CACAT CCTTGGCTCCGGATAGCTTCCAG	This paper	N/A
Reverse primer for mCherry-Rpb1 downstream homology arm: TCTGAT GCTTCCACTCGGCGGTGAGATC	This paper	N/A
Forward primer for EGFP-Rpb3 upstream homology arm: GTTTC AGAAGAGTGGGACATTTGGC	This paper	N/A
Reverse primer for EGFP-Rpb3 downstream homology arm: CAAAG GATCTACGAGACCGCACTGGA	This paper	N/A
Recombinant DNA		
pU6-BbsI-chiRNA	(Gratz et al., 2013)	Addgene plasmid #45946
pDsRed-attP	Gift from Melissa Harrison & Kate O’Connor-Giles & Jill Wildonger	Addgene plasmid #51019
pU6-Rpb1-Ntag	This paper	N/A
pU6-Rpb3-Ntag	This paper	N/A
pHD-mCherry-Rpb1	This paper	N/A
pHD-EGFP-Rpb3	This paper	N/A
Software and algorithms		
Volocity 6.3	Quorum	
FIJI (ImageJ)	(Schindelin et al., 2012)	
Python 3	Python Software Foundation	https://www.python.org
R v3.2.1	The R Foundation	https://www.r-project.org/

## Highlights

- Transcription and DNA replication overlap in early *Drosophila* embryos
- Transcripts elongating on unreplicated genes will clash with replication forks
- Transcripts beginning after replication follow forks and avoid collisions
- We show that post-mitotic transcription is coupled to replication in embryos
