# Supplementary material for: Coordinating transcription and replication to mitigate their conflicts in early Drosophila embryos
Source: Cell Rep. Author manuscript; Available in PMC 2022 Nov 16. (PMC9667882; doi:10.1016/j.celrep.2022.111507)
Supplement: 1 [file NIHMS1843693-supplement-1.pdf]

Cell Reports, Volume *41*

Supplemental information

Coordinating transcription and replication to mitigate their conflicts in early Drosophila embryos

Chun-Yi Cho, James P. Kemp Jr., Robert J. Duronio, and Patrick H. O'Farrell

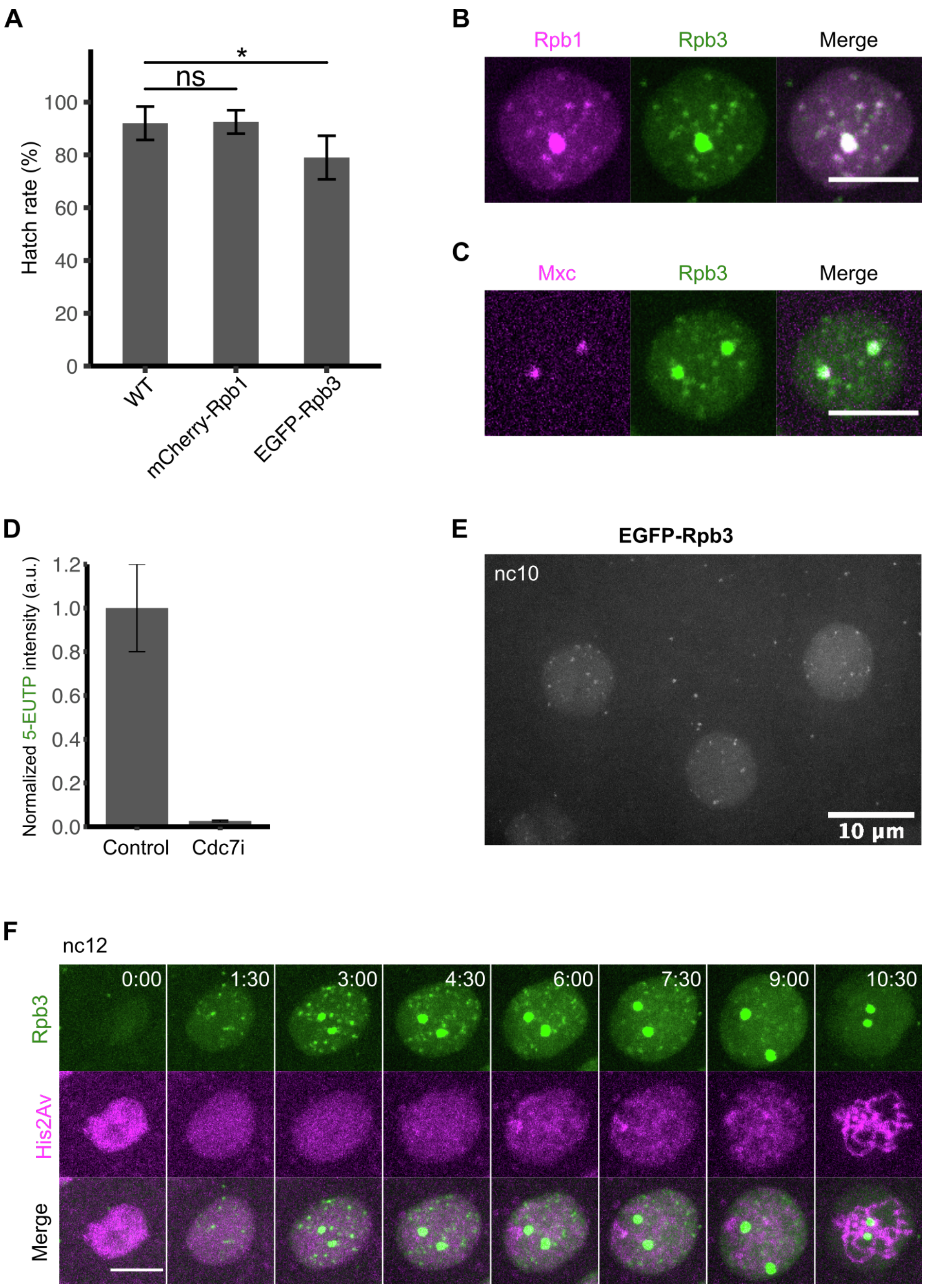

Figure S1, Related to Figure 1. Endogenously tagged RNA polymerase II subunits form two classes of clusters.

(A) Embryo hatch rates in wild type or fly lines carrying endogenously tagged RNA polymerase II (RNAPII) subunits. The genotypes indicate both that of mothers and embryos. Data are shown as mean ± s.d. (n = 4 independent experiments). *p<0.05 by one-sided Student's t-test.

(B, C) Confocal live imaging of EGFP-Rpb3 embryos co-expressing either mCherry-Rpb1 or Mxc-mScarlet during nuclear division cycles. Scale bars, 5 μm.

(D) Mean fluorescent intensity of 5-EUTP/Alexa488 in the nucleus in embryos injected with water as control or Cdc7i. See the legend for Figure 1D for more experimental details. Error bars represent s.d., n = 3 embryos.

(E) A snapshot of an embryo expressing EGFP-Rpb3 at nc10, when the nuclei first migrated to the surface of the embryo.

(F) Representative stills from live imaging of EGFP-Rpb3 and His2Av-RFP in an embryo during nc12. The timepoint 0 is set as the first frame when nuclei marked by His2Av changed into oval shape after mitosis. Scale bar, 5 μm.

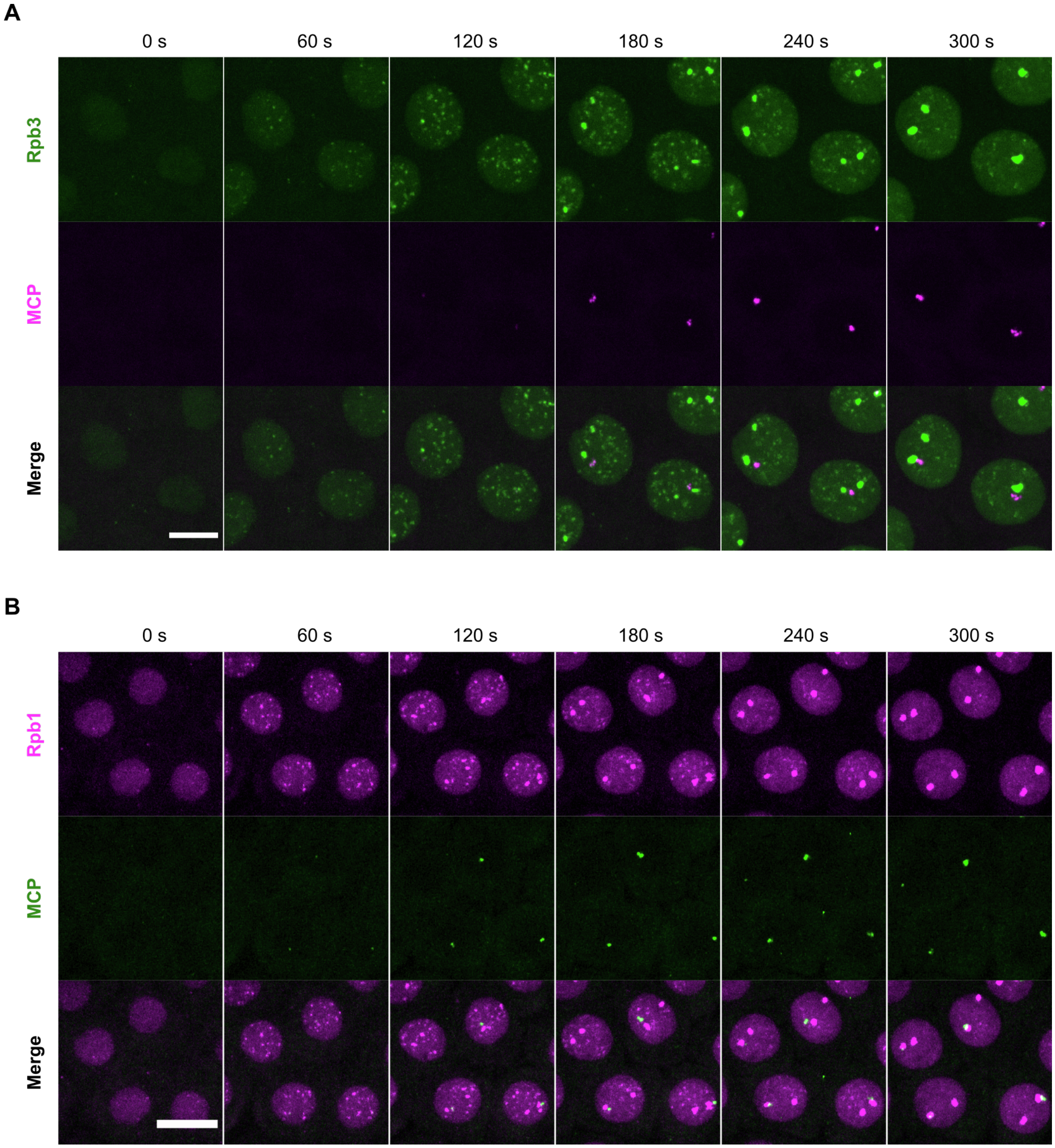

Figure S2, Related to Figure 2. Live imaging of RNAPII clusters in syncytial-blastoderm stage embryos of *Drosophila*.

(A) Representative stills from live imaging of EGFP-Rpb3 and MCP-mCherry in embryos carrying *hbP2-MS2* during nc12. Similar outcomes were observed in 10 embryos. Scale bar, 6 μm.

(B) Representative stills from live imaging of mCherry-Rpb1 and MCP-GFP in embryos carrying *hbP2-MS2* during nc12. Similar outcomes were observed in 4 embryos. Scale bar, 10 μm.

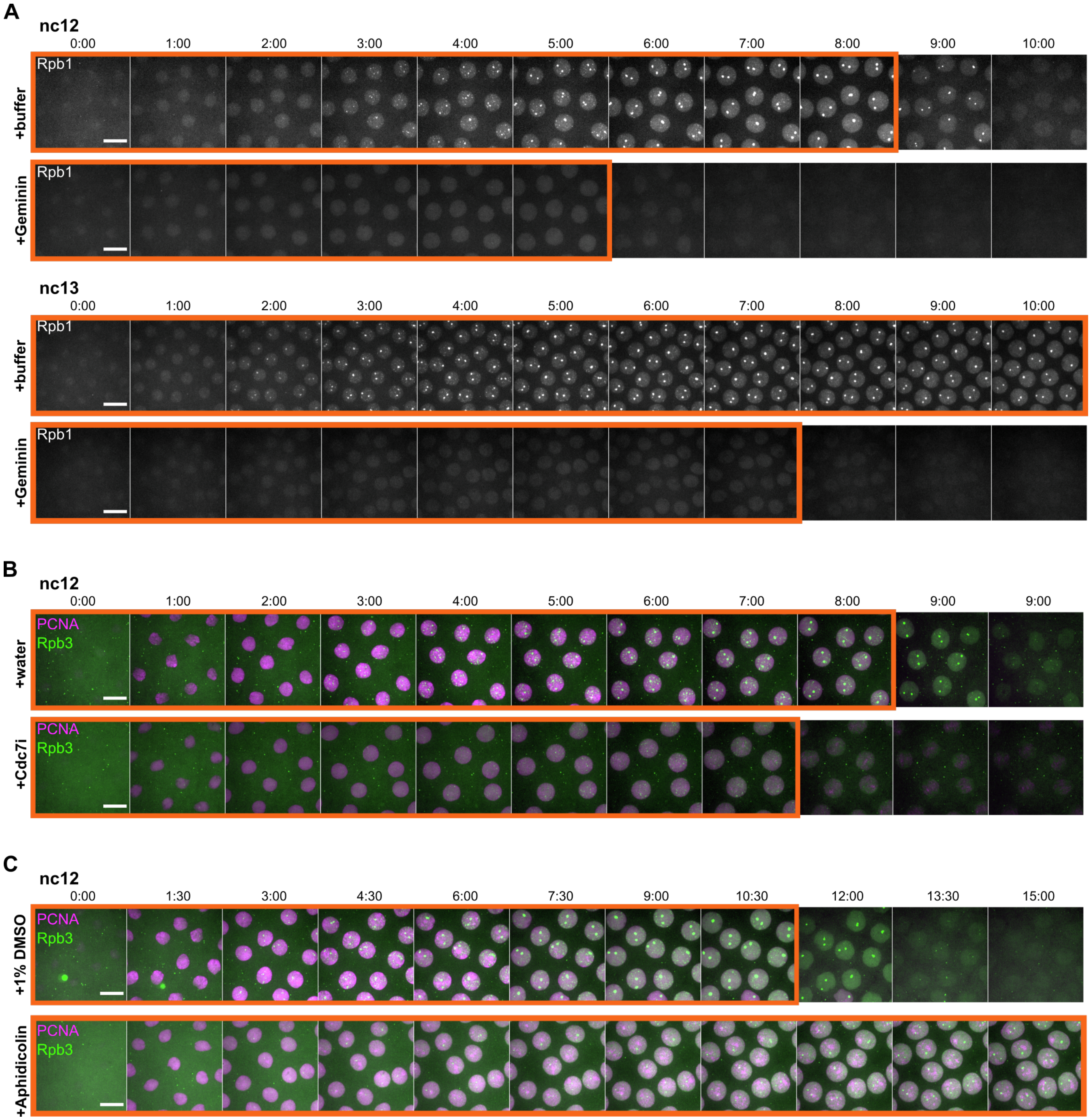

Figure S3, Related to Figure 3. Inhibiting DNA replication suppresses RNAPII clustering and changes interphase duration.

(A) Representative stills from live imaging of mCherry-Rpb1 in embryos injected with microinjection buffer as control or 20 mg/ml ECFP-Geminin. The 0-minute timepoint is set as the first frame with nuclear mCherry-Rpb1 signal upon entering interphase. Similar outcomes were observed in at least 3 embryos for each experiment.

(B, C) Representative stills from live imaging of mCherry-PCNA and EGFP-Rpb3 in embryos after indicated injection. The 0-minute timepoint is set as the first frame with visible chromatin-bound PCNA.

The orange boxes outline frames in interphase. All scale bars, 10 μm.
